# Perioperative pediatric mortality in Ethiopia: A prospective cohort study

**DOI:** 10.1016/j.amsu.2021.102396

**Published:** 2021-06-09

**Authors:** Fantahun Tarekegn, Rahel Seyoum, Gashaw Abebe, Misganew Terefe

**Affiliations:** Department of Anesthesia, Bahir Dar University, Ethiopia

**Keywords:** Perioperative, Mortality, Pediatric, Outcome, ASA, American Society of Anesthesiologists, CI, Confidence Interval, OR, Odds Ratio

## Abstract

**Background:**

There was recording of excellent outcomes for pediatric surgery in developed countries of the world when it was carried out by an experienced pediatric surgeon and anesthetists with availabilities of equipment. However, this circumstance was not the ordinary for developing countries. The main objective of our study was to launch a pediatric perioperative mortality rate reference point and determination of associated factors under general or regional anesthesia in Ethiopia.

**Materials and methods:**

the prospective electronic based data collection was done at Tibebe Ghion Specialized Teaching Hospital, Ethiopia with case specific of perioperative data for age less than 18 years old. We computed patients with mortality at 24 h, 48 h and 7 days in the form of percentages. Logistic regression was used for evaluation of mortality at different predictor variables.

**Results:**

from 849 cases analyzed, there were mortality rate of 0.59%, 1.42%, and 2.58% within 24 h, 48 h and 7 days of surgery, respectively. The emergency surgeries (OR = 2.80 [95% CI, 1.78–3.82]; p < 0.03) were associated with an increased risk of mortality within 7 days of post-surgery.

**Conclusion:**

Despite the progresses reached in the pediatric anesthesia and surgical safety in Tibebe Ghion Specialized Teaching Hospital, the pediatric perioperative mortality rates were still high or comparable to other low income African countries. Emergency surgeries were associated with an increased risk of perioperative mortality within 7 days of surgical intervention.

Tibebe Ghion Specialized Teaching Hospital should emphasis on evaluation and monitoring of outcome for reduction of mortality with the emergency surgeries younger than 18 years old. We also suggested doing this research work at larger sample sizes for more actual information.

## Highlights

• Perioperative mortality rate was a quality indicator of perioperative care.
• There was recording of excellent outcome for pediatric surgery.
• Pediatric perioperative mortality rates were still higher in low income African countries.
• Emergency surgeries were associated with an increased risk of pediatric perioperative mortality within 7 days of surgery.
• Pediatrics were more risk at emergent status than elective during perioperative period.

## 1 Background

In developing countries of the world, children are contained more than 50% of the population with younger than 18 years old. From these, it has been projected that 85% of the children possibly will requisite some kind of surgical intervention before their 15th years of adolescent period [1]. Surgical pediatric admission labeled with 6–12% of entire pediatric admission, even though numerous of their admissions were included to somewhat minor surgeries [2,3]. Unable to develop some surgical involvement package, possibly lead to lifelong disability, social exclusion or untimely death, normally prevented by appropriate intervention [4].

To measure the effectiveness of surgical interventions, there is an essential to establish a model reached with consensus of clinical outcomes and to observe changes as intervention are completed. ‘‘Perioperative mortality rate defined as the number of patients of all cases death before discharge divided by total number of procedures, and presented as percentage, is a quality indicator of perioperative care’’ [5].

The safe provision of anesthesia depends on preexisting condition of the child. However, the preoperative investigation availability is limited in rural areas of low and middle income countries [1]. Furthermore, the harmless practice of pediatric anesthesia is mainly related to appropriate sized face mask, tracheal tubes, oropharyngeal airways, laryngoscope and intravenous cannula, but there is less accessibility of these crucial equipment [6]. The most common practice of anesthetic in numerous rural areas of district hospitals is ketamine [7]. In addition, halothane leftovers with the mainstay of volatile agent in anesthesia practice of Africa [8].

There was recording of excellent outcomes for pediatric surgery when it was carried out by an experienced pediatric surgeon and anesthetists with availabilities of equipment [8,9]. However, this circumstance was not the ordinary for developing countries of the world. This could be due to the sum effects of deprived overall health of the children, not earlier presentation, and anesthetic and surgical complications, commonly resulted through poor outcomes when compared to developed countries [10]. The perioperative pediatric mortality in Africa was ranged from less than 3% for cautiously chosen cases to 50% for emergency neonatal bowel obstruction surgery even though there has been limited accessibility of data information [11]. According to a meta-analysis study between 2005 to 2014 years, there was recorded of 29.4% perioperative pediatric mortality in African countries (11). A Kenyan study showed that the pediatric perioperative mortality rate was 0.8%, 1.1%, 1.7% at 24 h, 48 h and 7 day of post-surgery, respectively [12]. The main objective of our study was to launch a pediatric perioperative mortality rate reference point and determination of associated factors under general or regional anesthesia in Ethiopia.

## 2 Materials and Methods

### 2.1 Study design

This prospective cohort study was approved by Ethical Review Board of Bahir Dar University College of Medicine and Health Sciences with waived of informed consent. The study was also registered retrospectively in research registry with a research registry unique identifying number (UIN) of researchregistry6531. Our data collection was employed from August 2018 to August 2020 with having anonymous identity. This study has been conducted in line to strengthening the reporting of cohort studies in surgery (STROCSS) criteria [13].

### 2.2 Inclusion/exclusion criteria

#### 2.2.1 Inclusion criteria

We included our data collection with all pediatric (age less than 18 years old) patients who were scheduled for surgical intervention at Tibebe Ghion Specialized Teaching Hospital.

#### 2.2.2 Exclusion criteria

We did not consider the data collection when those patients were missed the prospective follow up study.

### 2.3 Variables of the study

#### 2.3.1 Dependent variables

The outcome variables of our study were pediatric perioperative mortality at 24 h, 48 h and 7 days of post-surgery. The time to death was considered as the number of hours from patient's date of surgery to the date of death (rounded to nearest hours or day). The mortality variables were formed for three time periods after surgery, 24 h, 48 h and 7 days. Mortality was also measured as a binary variable (0 if died, and 1 if did not die).

#### 2.3.2 Independent variables

The independent variables of our study were American Society of Anesthesiologists (ASA) physical status, age, type of procedures, and urgency of surgery.

### 2.4 Data collection

The data collection was done by anesthetists, who were working at Tibebe Ghion Specialized Teaching Hospital. They performed the filling of data through the already prepared electronic based data collection system tools. The data collectors (anesthetists and anesthesia students) were trained about data collection and how the system was working to the respective variables of patient demographics and clinical characteristics, including perioperative mortality data at 24 h, 48 h, and 7 days of post-surgery. There was one data manager to control the overall data collection with double check of the patients’ outcome and also information technology (IT) manager to check the functionality of the system on tablets per 24 h round. The data was collected from August 2018 to August 2020.

The data collectors followed patients’ outcome with presence of mortality, starting from preoperative assessment to 7 days of post-surgery, prospectively. Furthermore, the data manager controlled the filling of data and having telephone call of each patient if they discharged earlier than 7 days of surgery. The impatient mortality was confirmed through the hospital logbook with double check by data manager.

### 2.5 Statistical analysis

A convenient sample size was used instead of power calculation. However, we tried to use the maximum number of patients during the study period. We used stata (version 14) for data analysis. The data was treated with each occurrence of deaths as an individual end point (coded as a binary variable, 0 or 1). Patient demographics, clinical characteristics, and outcomes were summarized using mean ± SD or median (IQR) for continuous variables and percentage for categorical variables. Logistic regression was used to show the association of outcome to independent variables of the study. P value < 0.05 was considered as a significant result.

## 3 Results

**Fig. 1:**
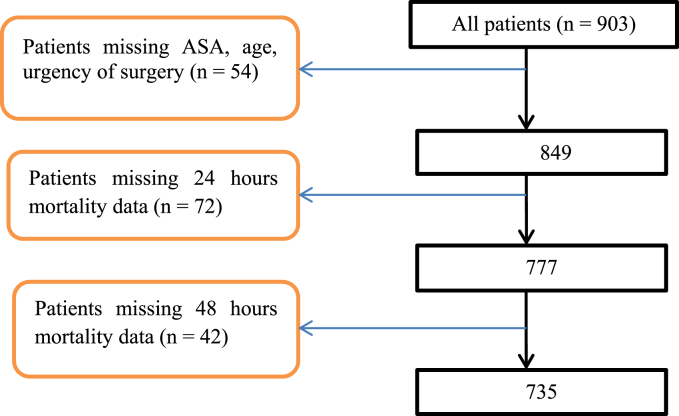
The number of cases with missing data at Tibebe Ghion Specialized Teaching Hospital.

The data were collected from August 2018 to August 2020. This has been completed over 903 cases at Tibebe Ghion Specialized Teaching Hospital through electronic based data collection system. The 54 (%) of patients were excluded with missing of ASA, age and urgency of surgery and 849 cases entered to 24 h postoperative mortality analysis (Fig. 1). Table 1 showed the details of patients and case demographic data. In this table, about 93% of the cases were completed by safe surgery checklist. The mortality rate was estimated as 0.59%, 1.42%, and 2.58% within 24 h, 48 h and 7 days of surgery, respectively. The emergency surgeries were associated with an increased risk of 7 days of mortality at Table 2. However, there was not a significant association to demographic and clinical characteristic variables (Table 1).

**Table 1:** Patient demographics and clinical characteristic.

Variables	Number (%)
Total number of patients	849
ASA Classification	
ASA I	598 (70.43%)
ASA II	211 (24.85%0
ASA III	31 (3.65%)
ASA IV	5 (0.59%)
ASA V	4 (0.47%)
Sex	
Male	533 (62.78%)
Female	316 (37.22%)
Age	
<1 month	109 (12.84%)
1–12 months	133 (15.66%)
1–5 years	171 (20.14%)
6–10 years	155 (18.26%)
11^+^	281 (33.10%)
Anesthesia type	
General	652 (76.80%)
Spinal	197 (23.20%)
Safe surgery checklist used	787 (92.70%)
Patient district	
Awi	41 (4.82%)
East Gojjam	184 (21.67%)
North Gondar	70 (8.24%)
South Gondar	113 (13.31%)
Wello	17 (2.00%)
West Gojjam	424 (49.94%)
Emergency cases	445 (52.41%)
Trauma cases	248 (29.21)
Blood transfused	49 (5.81%)

**Table 2:** Perioperative mortality at 24 h, 48 h and 7 days of post-surgery.

Type of surgery cases	24 h	OR (95%,CI)	P -value	48 h	OR (95%,CI)	P -value	7 days	OR (95%,CI)	P -value
Total surgical cases	849			777			735		
Total number of deaths (%)	5/849 (0.59%)			11/777 (1.42%)			19/735 (2.58%)		
Elective	3/849 (0.35%)	0.67 (0.32–2.06)	0.08	5/777 (0.65%)	1.2 (0.01–2.38)	0.06	5/735 (0.68%)	2.8 (1.78–3.82)	0.03
Emergency	2/849 (0.24%)			6/777 (0.77%)			14/735 (1.90%)		

% is the percent of respective variables with not including the missing cases, CI = Confidence Interval.

## 4 Discussion

Perioperative mortality rate was the vital for evaluating the quality of care, safety of anesthesia and surgery, decisively to the low income countries where the accessibility of anesthesia and surgery facilities were limited [14]. The main purpose of this study was to launch a pediatric perioperative mortality rate reference point and determination of associated factors under general or regional anesthesia in our country, Ethiopia. So, the study demonstrated that the perioperative mortality rate of pediatrics with age younger than 18 years old were 0.59%,1.42% and 2.58% at 24 h, 48 h and 7 days of post-surgery, respectively. This was the lowered magnitude of mortality compared to Kenyan study, except for the higher percentage of deaths recorded at 7 days of post-surgery [12]. In addition, it was smaller than other studies of low-income countries of Africa [15], but still 100 to 300 times more than pediatric perioperative mortality in high resource sites [11,16,17].

Our study does not clarify why the lowered perioperative mortality rates were documented in our setting compared to most African countries. However, we can put forward three possible reasons. First, large numbers of cases were done or consulted by pediatric surgeons and experienced master anesthetists. Second, the hospital had monitors, such as pulse oximeter designed for children and blood pressure cuffs. Third, Tibebe Ghion Specialized Teaching Hospital had a system that focuses a standardized care protocol of two anesthetists per operation theatre for any child surgery, even though there was higher man power shortage and poor facilities faced to the low income countries of Africa [18]. Furthermore, the 92.70% of cases were done through filling of safe surgery checklists (Table 2). This activity was completed only to 25% in east African countries (Uganda, Tanzania, Kenya, Rwanda, and Burundi) even though 58% of anesthesia providers familiarized with the checklists [19]. In contrast, there was an enhanced result for the reduction of perioperative complication rates in high income countries through the implementation of safe surgery checklists [20].

The logistic regression model to predictors of pediatric perioperative mortality disclosed that the emergency surgeries (OR = 2.80 [95% CI, 1.78–3.82]; p < 0.03) were associated with an increased risk of perioperative mortality within 7 days of surgical intervention. This was supported by other studies [[21], [22], [23], [24]] which stated that patients’ perioperative mortality were more risk at emergent status than other elective cases after surgery.

There were some limitations in our study. First; the sample sizes were smaller. Second: It was a single center study. Third: the study was not demonstrating the outcome with consideration of each surgical departments or surgical types.

## 5 Conclusion

Despite the progresses achieved in the pediatric anesthesia and surgical safety in Tibebe Ghion Specialized Teaching Hospital, the pediatric perioperative mortality rates were still high or comparable to other low income African countries. Emergency surgeries were associated with increased risk of perioperative mortality within 7 days of surgery.

Tibebe Ghion Specialized Teaching Hospital should emphasis on evaluation and monitoring of outcome for reduction of mortality with the emergency surgeries younger than 18 years old. We also suggested doing this research at larger sample sizes for more actual information.

## Ethical approval

The ethical approval was given by College of Medicine and Health Sciences, Bahir Dar University ethical review committee.

## Source of funding

We received a grant from Impact Africa Project.

## Author contribution

1. Fantahun Tarekegn: make substantial contributions to conception and design, and/or acquisition of data, and/or analysis and interpretation of data.

2. Misganew Terefe: participate in drafting the article or revising it critically for important intellectual content; and.

3. Gashaw Abebe: give final approval of the version to be submitted and any revised version.

## Registration of research studies

1.Name of the registry: Research Registry.

2.Unique Identifying number or registration ID: researchregistry6531.

3.Hyperlink to your specific registration (must be publicly accessible and will be checked): https://www.researchregistry.com/.

## Guarantor

Fantahun Tarekegn.

## Consent

Bahir Dar University, Tibebe Ghion Specialized Teaching Hospital approved the study to be conducted without involvement of written informed consent.

## Provenance and peer review

Not commissioned, externally peer reviewed.

## Declaration of competing interest

All authors declared that we have not any competing of interests.
